# Phase 1 study of ixazomib, an investigational proteasome inhibitor, in advanced non-hematologic malignancies

**DOI:** 10.1007/s10637-015-0230-x

**Published:** 2015-03-18

**Authors:** David C. Smith, Thea Kalebic, Jeffrey R. Infante, Lillian L. Siu, Daniel Sullivan, Gordana Vlahovic, John S. Kauh, Feng Gao, Allison J. Berger, Stephen Tirrell, Neeraj Gupta, Alessandra Di Bacco, Deborah Berg, Guohui Liu, Jianchang Lin, Ai-Min Hui, John A. Thompson

**Affiliations:** 1 University of Michigan Comprehensive Cancer Center, 7302 CC SPC 5948, 1500 E. Medical Center Drive, Ann Arbor, MI 48109-5948 USA; 2 Takeda Pharmaceuticals International Co., Cambridge, MA USA; 3 Sarah Cannon Research Institute, Nashville, TN USA; 4 Princess Margaret Hospital, Toronto, ON Canada; 5 H. Lee Moffitt Cancer Center and Research Institute, Tampa, FL USA; 6 Duke Comprehensive Cancer Center, Durham, NC USA; 7 Winship Cancer Institute of Emory University, Atlanta, GA USA; 8 Seattle Cancer Care Alliance, Seattle, WA USA

**Keywords:** 20S proteasome, Activating transcription factor 3, Ixazomib, Non-hematologic malignancies, Phase 1 clinical trial (4–6)

## Abstract

*Purpose* Ixazomib is an investigational proteasome inhibitor with demonstrated antitumor activity in xenograft models of multiple myeloma (MM), lymphoma, and solid tumors. This open-label, phase 1 study investigated intravenous (IV) ixazomib, in adult patients with advanced non-hematologic malignancies. *Methods* Patients received IV ixazomib twice-weekly for up to twelve 21-day cycles. The 0.125 mg/m^2^ starting dose was doubled (one patient/dose) until 1.0 mg/m^2^ based on dose-limiting toxicities (DLTs) in cycle 1. This was followed by 3 + 3 dose-escalation and expansion at the maximum tolerated dose (MTD). Primary objectives included safety and MTD assessment. Secondary objectives included assessment of pharmacokinetics, pharmacodynamics, and disease response. *Results* Ixazomib was escalated from 0.125 to 2.34 mg/m^2^ to determine the MTD (*n =* 23); patients were then enrolled to MTD expansion (*n* = 73) and pharmacodynamic (*n* = 20) cohorts. Five patients experienced DLTs (1.0 and 1.76 mg/m^2^: grade 3 pruritic rash; 2.34 mg/m^2^: grade 3 and 4 thrombocytopenia, and grade 3 acute renal failure); thus, the MTD was 1.76 mg/m^2^. Drug-related grade ≥3 adverse events (AEs) included thrombocytopenia (23 %), skin and subcutaneous (SC) tissue disorders (16 %), and fatigue (9 %). Among 92 evaluable patients, one (head and neck cancer) had a partial response and 30 had stable disease. Ixazomib terminal half-life was 3.8–7.2 days; plasma exposures increased dose-proportionally and drug was distributed to tumors. Inhibition of whole-blood 20S proteasome activity and upregulation of ATF-3 in tumor biopsies demonstrated target engagement. *Conclusions* In patients with solid tumors, ixazomib was associated with a manageable safety profile, limited antitumor activity, and evidence of downstream proteasome inhibition effects.

**Electronic supplementary material:**

The online version of this article (doi:10.1007/s10637-015-0230-x) contains supplementary material, which is available to authorized users.

## Introduction

The ubiquitin-proteasome system is a key pathway for protein degradation and as such plays a key role in protein homeostasis [1, 2]. Clinically, bortezomib has shown proteasome inhibition to be a highly promising treatment strategy for multiple myeloma (MM) and other hematologic malignancies [3–6]. Despite the proven efficacy in hematologic malignancies, clinical activity is limited in solid tumors [7–13]. Preclinical data; however, implied the potential for antitumor effects in solid tumor models [14–18]. It has been suggested that a lack of drug penetration into the tumor may be the cause of this modest antitumor activity [19, 20].

The investigational agent ixazomib is an orally bioavailable, small molecule, potent, reversible, and selective inhibitor of the β5 site of the 20S proteasome, which is structurally different from bortezomib [20]. Ixazomib is currently being investigated in phase 3 trials in MM (Clinicaltrials.gov identifiers NCT01564537 and NCT01850524) and primary systemic AL amyloidosis (NCT01659658). When compared with bortezomib, ixazomib demonstrated faster dissociation from the proteasome in vitro, and data from mouse xenograft models of human cancers suggested increased proteasome inhibition in tumor tissue [20]. Preclinical studies also demonstrated the antiproliferative activity of ixazomib in tumor cell lines with potent antitumor activity in xenograft models of MM, lymphoma, and some solid tumors [20–22]. These data provided the rationale for the clinical development of ixazomib in both hematologic and non-hematologic malignancies. In clinical trials conducted to date, ixazomib was studied as an intravenous (IV) formulation, initially, and subsequently as an oral formulation.

This paper reports the results of the first-in-human, phase 1 dose-escalation study (NCT00830869) of twice-weekly IV ixazomib in patients with advanced solid tumors. The study included a drug distribution and pharmacodynamic assessment of activating transcription factor-3 (ATF-3) levels in solid tumor tissue as a marker of target engagement and an evaluation of whole blood 20S proteasome activity.

## Materials and methods

### Patients

Eligible participants were aged ≥18 years with a diagnosis of a non-hematologic malignancy for which standard treatment was no longer effective or did not offer curative or life-prolonging potential. Eastern Cooperative Oncology Group (ECOG) performance status of 0–2, absolute neutrophil count (ANC) ≥1,500/mm^3^, platelet count ≥100,000/mm^3^, total bilirubin ≤1.5 × the upper limit of normal (ULN), alanine aminotransferase (ALT) and aspartate aminotransferase (AST) ≤2.5 × ULN (≤5 × ULN if the elevation can be reasonably ascribed to metastatic disease), creatinine clearance or calculated creatinine clearance ≥60 mL/min, and QTc <470 ms on 12-lead electrocardiogram were required. Patients also had no: peripheral neuropathy (PN) of grade ≥2; diarrhea of grade >1; recent (<14 days) major surgery; infection requiring systemic antibiotic therapy; symptomatic brain metastasis; uncontrolled cardiovascular conditions within the past 6 months; radiotherapy or systemic antineoplastic therapy within 21 days; or any investigational products within 28 days before the first dose. In the disease-specific expansion and tumor pharmacodynamic expansion (TPEC) cohorts, radiographically or clinically evaluable tumor (clinically measurable disease per Response Evaluation Criteria in Solid Tumors [RECIST]) was required. An elevated prostate specific antigen (PSA) level alone per modified Prostate Cancer Working Group 2 (PCWG2) criteria was acceptable for evaluation of prostate cancer.

All patients provided written informed consent and were free to withdraw at any time during the study. Institutional Review Boards at all participating institutions approved the study and the informed consent documents. The study was conducted according to the provisions of the Declaration of Helsinki, the International Conference on Harmonization, and Guidelines for Good Clinical Practice.

### Study design

This open-label, non-randomized, dose-escalation phase 1 study was conducted at 7 sites in the United States and Canada, with patients enrolled from March 02, 2009 to January 10, 2012. The primary objective of the trial was to determine the safety profile, establish the maximum tolerated dose (MTD), and inform the recommended phase 2 dose of IV ixazomib in patients with non-hematologic malignancies. Secondary objectives were to characterize the pharmacokinetics and pharmacodynamics of IV ixazomib in blood and to evaluate disease response. Drug distribution and target engagement in post-dose tumor samples was measured by determining the increase in ATF-3 levels in post-dose tumor samples compared with pre-dose samples by immunohistochemistry (IHC).

Patients received ixazomib as an IV bolus on days 1, 4, 8, and 11 of a 21-day cycle, for up to 12 cycles, or until disease progression or unacceptable toxicity. Patients who were thought to potentially benefit from prolonged therapy could receive continued treatment beyond 12 cycles according to the treating physician’s consideration. Patients could receive prophylactic antiviral therapy; prophylactic antidiarrheals and antiemetics were not employed initially but could be considered upon development of diarrhea or nausea/vomiting.

The dose-escalation cohort comprised patients with any non-hematologic malignancy. The four MTD expansion cohorts were limited to patients with non-small cell lung cancer (NSCLC), head and neck cancer (squamous cell cancer), soft tissue sarcoma, and prostate cancer, respectively. In addition, the TPEC was open to patients with non-hematologic malignancy that could be safely biopsied. Dose escalation initially proceeded from a starting dose of 0.125 mg/m^2^ of body surface area, with one patient per dose level and dose doubling up to a dose of 1.0 mg/m^2^. Dose doubling was permitted until one of the following occurred: one patient experienced a dose-limiting toxicity (DLT) during cycle 1; any two patients experienced a drug-related adverse event (AE) of grade ≥2 during cycle 1; or the dose level of 1 mg/m^2^ had been evaluated. Dose escalation then proceeded in 33 % increments following a standard 3 + 3 schema based on the occurrence of DLTs in cycle 1. DLTs were defined as any of the following: grade 4 neutropenia or thrombocytopenia lasting for >7 consecutive days, or a platelet count of <10,000/mm^3^ at any time; grade 3 neutropenia with infection/fever or thrombocytopenia with clinically significant bleeding; grade ≥3 PN; grade 3 QTc prolongation; any other grade ≥3 non-hematologic toxicity (despite optimal antiemetic prophylaxis for nausea/emesis and supportive therapy for diarrhea), except grade 3 arthralgia/myalgia or short-term (<1 week) grade 3 fatigue; a delay of >1 week in commencing cycle 2 due to lack of adequate recovery of ixazomib-related toxicities; or other grade ≥2 ixazomib-related toxicities requiring discontinuation in the opinion of the investigator. The MTD was defined as the highest dose level at which 0/3 or 1/6 patients experienced DLTs during cycle 1. Once the MTD had been established, patients (including those from the dose-escalation cohort meeting the eligibility criteria) were enrolled into four MTD expansion cohorts and the TPEC, as defined above.

### Assessments

AEs were monitored throughout the study and were graded according to National Cancer Institute Common Terminology Criteria for Adverse Events (NCI-CTCAE) v3.0. Response assessments were undertaken by investigators using a consistent imaging modality in accordance with RECIST 1.0 [23] on days 18–21 of cycle 2 and subsequent cycles. For patients with prostate cancer, response and progressive disease were based on RECIST for measurable neoplastic disease [23] or on measurement of serum PSA per modified PCWG2 criteria in the absence of measurable sites [24].

Blood samples for determination of plasma ixazomib concentrations for pharmacokinetic analysis (3 mL) and for measurement of whole blood 20S proteasome activity (1 mL) were collected at the following timepoints for all patients in the dose-escalation cohort and for the first 12 patients completing cycle 1 while receiving protocol-specified treatment at the MTD: pre-dose (within 1 h) on days 1, 4, 8, and 11 of cycle 1, and on day 1 of cycle 2; and on days 1 and 11, cycle 1, at 5, 15 and 30 min, and 1, 2, 4, 9, 24, 48, 96 (day 11 only), 120 (day 11 only), and 168 (day 11 only) h post-dose. A limited sampling schedule was employed for the remaining patients in the MTD expansion cohorts.

Plasma concentrations of ixazomib were measured using a good laboratory practice-validated liquid chromatography/tandem mass spectrometry (LC/MS/MS) assay with a lower limit of quantification of 0.5 ng/mL. Whole blood 20S proteasome activity (expressed as percent proteasome inhibition relative to baseline) was measured using an established fluorogenic assay [25], which evaluates the chymotrypsin-like activity of the β5 subunit of the 20S proteasome, the primary target of ixazomib.

Tumor biopsies were analyzed for the presence of ixazomib using a quantified LC/MS/MS methodology validated in xenograft models. Expression of ATF-3 was measured in the TPEC using an IHC staining assay developed and validated using preclinical and human primary tumor samples. For this assay, formalin-fixed, paraffin-embedded tumor biopsy samples were obtained pre- and post-treatment (at 4–24 h post-dosing on day 1 or day 4 of cycle 1). Six 5 μm sections from each tumor sample were then cut (10 μm apart) and stained with antibody to ATF-3 (Santa Cruz Biotechnology, Inc., sc-188, lot:K1908) on a Discovery XT autostainer (Ventana Medical Systems, Inc.). After staining, whole slides were scanned and analyzed to calculate the percentage ATF-3-positive area for each section using the Genie™ (Aperio® ePathology Solutions) pattern recognition tool. The ATF-3-positive area was measured within the tumor region using the Positive Pixel Count algorithm, and the percentage ATF-3-positive area/total tumor area was then calculated for each section.

### Statistical analyses

The safety population included all patients who received ≥1 dose of ixazomib, and the DLT-evaluable population, which was used for determination of the MTD, comprised patients who received all cycle 1 doses of ixazomib or experienced a DLT in cycle 1. The pharmacokinetic and pharmacodynamic populations included all patients in the dose-escalation cohort and the first 12 patients treated at the MTD who did not receive any excluded concomitant medications during the time of blood sampling, and who had sufficient concentration–time or effect–time data to permit reliable estimation of pharmacokinetic/pharmacodynamic parameters. The response-evaluable population included all patients who received ≥1 dose of ixazomib, had measurable disease at baseline, and had ≥1 post-baseline disease assessment. Approximately 16 response-evaluable patients were planned to be evaluated in each MTD expansion cohort. The binomial probability calculation showed that if the overall response rate was 10 %, there was approximately an 81 % probability of observing ≥1 response and a 49 % probability of observing ≥2 responses in 16 patients.

All efficacy and safety data were summarized using descriptive statistics. Pharmacokinetic and pharmacodynamic parameters were calculated using noncompartmental methods (WinNonlin software v5.3).

Approximately 16 patients were planned to be enrolled to the TPEC to estimate ixazomib concentrations in tumors and target engagement as ATF-3 expression levels. A survey of human tumors on tissue microarrays, some with multiple tumor samples derived from the same individual, was performed to evaluate between-subject and within-subject variability in the ATF-3-positive area. A mixed-effect model using data from all evaluable pre- and post-dose samples estimated a within-subject coefficient of variation of 28.33 %. This value was used to determine the minimum number of sections (*n* = 6) for pharmacodynamic analysis and the size of the fold change in ATF-3 that could be detected with 80 % power. Student’s t-tests on individual patient data were used to determine the number of patients with a significant fold increase (*p* < 0.05) in ATF-3 after ixazomib treatment.

## Results

### Patients

A total of 116 patients were enrolled in this study. Twenty-three patients were enrolled to the dose-escalation cohort, including: one patient each at 0.125, 0.25, and 0.5 mg/m^2^; seven patients at 1 mg/m^2^; four patients at 1.33 mg/m^2^; six patients at 1.76 mg/m^2^; and three patients at 2.34 mg/m^2^. Seventy-three patients were enrolled to the MTD expansion cohorts, including 20 with NSCLC, 22 with head and neck cancer, 20 with soft tissue sarcoma, and 11 with prostate cancer. A total of 20 patients were enrolled to the TPEC.

Patients’ demographics and baseline disease characteristics are summarized in Table 1. Patients had received a median of 3 prior lines of therapy (range, 1 to > 4) and median time since primary diagnosis among all patients was 2.5 years.

**Table 1:** Patients’ demographics and baseline disease characteristics

Characteristic	Dose-escalation cohort^a^ (*n* = 23)	MTD expansion cohorts (*n* = 93)					Total (*N* = 116)
		NSCLC (*n* = 20)	H&N (*n* = 22)	STS (*n* = 20)	PC (*n* = 11)	TPEC^b^ (*n* = 20)	
Median age, years (range)	60.0 (37–79)	60.5 (35–80)	56.5 (29–72)	56.5 (34–73)	64.0 (55–73)	55.5 (34–78)	58.0 (29–80)
Male, *n* (%)	9 (39)	10 (50)	18 (82)	8 (40)	11 (100)	10 (50)	66 (57)
Race, *n* (%)							
White	21 (91)	17 (85)	18 (82)	17 (85)	10 (91)	12 (60)	95 (82)
African-American	1 (4)	1 (5)	2 (9)	2 (10)	1 (9)	7 (35)	14 (12)
Asian	0	2 (10)	2 (9)	1 (5)	0	1 (5)	6 (5)
Other	1 (4)	0	0	0	0	0	1 (<1)
ECOG performance status, *n* (%)							
0	9 (39)	9 (45)	6 (27)	11 (55)	5 (45)	8 (40)	48 (41)
1	14 (61)	11 (55)	11 (50)	7 (35)	6 (55)	11 (55)	60 (52)
2	0	0	5 (23)	2 (10)	0	1 (5)	8 (7)
Median time since primary diagnosis, years (range)	3.1 (0.8–12.1)	2.6 (0.6–6.8)	1.8 (0.7–19.6)	2.4 (0.3–12.5)	5.1 (1.3–18.5)	2.1 (0.3–19.8)	2.5 (0.3–19.8)
Number of prior lines of therapy, *n* (%)							
1	2 (9)	0	3 (14)	5 (25)	0	3 (15)	13 (11)
2	5 (22)	3 (15)	3 (14)	5 (25)	2 (18)	4 (20)	22 (19)
3	5 (22)	4 (20)	7 (32)	1 (5)	1 (9)	7 (35)	25 (22)
4	3 (13)	6 (30)	4 (18)	4 (20)	4 (36)	1 (5)	22 (19)
≥ 5	8 (35)	7 (35)	5 (23)	3 (15)	4 (36)	5 (25)	32 (28)
Prior radiation, *n* (%)	11 (48)	13 (65)	19 (86)	12 (60)	8 (73)	7 (35)	70 (60)

^a^Primary diagnoses included colon/colorectal cancer (*n* = 7), skin cancer (*n* = 4), kidney/renal cancer (*n* = 3), STS (*n* = 3), pancreatic cancer (*n* = 2), PC (*n* = 2), small-cell lung cancer (*n* = 1), and thyroid cancer (*n* = 1)

^b^Primary diagnoses included colon/colorectal cancer (*n* = 8), liver cancer (*n* = 2), adrenal, kidney, H&N, common bile duct, esophageal, ovarian, rectal cancer, melanoma, STS, and undifferentiated carcinoma (each *n* = 1)

*ECOG* Eastern Cooperative Oncology Group, *H&N* head and neck cancer, *MTD* maximum tolerated dose, *NSCLC* non-small cell lung cancer, *PC* prostate cancer, *STS* soft tissue sarcoma, *TPEC* tumor pharmacodynamic expansion cohort

### DLTs and determination of MTD

Of the 23 patients enrolled in the dose-escalation phase, 22 received all doses of ixazomib during cycle 1 and either completed the cycle or developed a DLT during the cycle; these 22 patients were included in the DLT-evaluable population. One patient died from progressive thyroid cancer and did not receive their day 11 dose, and hence was not DLT-evaluable.

Five patients experienced DLTs. One patient treated at the 1.0 mg/m^2^ dose level reported a DLT of grade 3 pruritic rash. Ixazomib dosing was held for this patient and, following administration of concomitant medication, the rash resolved within 10 days and the patient continued at a lower dose. At the 1.76 mg/m^2^ dose level, one patient reported a DLT of grade 3 pruritic rash, which persisted despite reducing and holding the dose of ixazomib; therapy was subsequently discontinued. The patient was treated with hydroxyzine, methylprednisolone, and diphenhydramine, and the pruritic rash resolved after 42 days. DLTs reported in three patients treated at the ixazomib 2.34 mg/m^2^ dose level were: grade 4 thrombocytopenia; grade 3 thrombocytopenia with grade 1 rectal hemorrhage; and grade 3 acute renal failure (pre-renal azotemia associated with nausea, vomiting, diarrhea, and dehydration). The patient with grade 4 thrombocytopenia was hospitalized, and the ixazomib dose was delayed and reduced. The patient with grade 3 thrombocytopenia with grade 1 rectal hemorrhage was admitted to hospital and subsequently died due to progressive disease before the next dose of study drug was to be administered. The patient with grade 3 acute renal failure was hospitalized and ixazomib was permanently discontinued. The MTD of ixazomib was thus determined to be 1.76 mg/m^2^ administered on days 1, 4, 8, and 11 of a 21-day cycle. Patients enrolled to the MTD expansion cohorts and the TPEC were treated at this dose of ixazomib.

### Treatment exposure and safety profile

Patients received a median of 2 treatment cycles (range, 1 to 12) overall, and across all individual cohorts. The maximum number of cycles received varied by cohort: the maximum number of cycles was 10, 8, 12, 7, 4, and 4 cycles in the dose-escalation, NSCLC, head and neck cancer, soft tissue sarcoma, and prostate cancer cohorts, and the TPEC, respectively. Overall, 23 patients (20 %) received ≥4 cycles; 22 of 99 patients (22 %) treated at the MTD received ≥4 cycles of therapy. Mean ixazomib dosing compliance (percent total dose received/total dose expected during time on treatment) was 97.9 % overall, and was similar across cohorts.

All 116 patients received ≥1 dose of ixazomib and were included in the safety population. Of these patients, 115 (99 %) experienced ≥1 treatment-emergent AE and 104 (90 %) experienced ≥1 drug-related AE (Supplementary Table 1). The most common drug-related AEs are summarized in Table 2. A total of 84 patients (72 %) had ≥1 treatment-emergent grade ≥3 AE; 66 patients (57 %) had ≥1 drug-related grade ≥3 AE. The most common drug-related grade ≥3 AEs are shown in Table 3.

**Table 2:** The most common (≥10 % of patients overall) drug-related AEs, overall and within the dose-escalation and expansion cohorts

AE, *n* (%)	Dose-escalation cohort (*n* = 23)	MTD expansion cohorts (*n* = 93)					Total (*N* = 116)
		NSCLC (*n* = 20)	H&N (*n* = 22)	STS (*n* = 20)	PC (*n* = 11)	TPEC (*n* = 20)	
Skin and SC tissue disorders^a^	7 (30)	9 (45)	12 (55)	12 (60)	9 (82)	12 (60)	61 (53)
Fatigue	8 (35)	11 (55)	11 (50)	8 (40)	7 (64)	10 (50)	55 (47)
Thrombocytopenia	6 (26)	8 (40)	11 (50)	8 (40)	6 (55)	13 (65)	52 (45)
Nausea	7 (30)	8 (40)	9 (41)	4 (20)	4 (36)	9 (45)	41 (35)
Decreased appetite	7 (30)	6 (30)	5 (23)	5 (25)	4 (36)	11 (55)	38 (33)
Vomiting	5 (22)	8 (40)	7 (32)	6 (30)	2 (18)	8 (40)	36 (31)
Diarrhoea	4 (17)	6 (30)	7 (32)	5 (25)	0	5 (25)	27 (23)
Peripheral neuropathies NEC^b^	2 (9)	2 (10)	1 (5)	5 (25)	3 (27)	4 (20)	17 (15)
Pyrexia	0	5 (25)	2 (9)	5 (25)	0	4 (20)	16 (14)
Stomatitis	0	3 (15)	3 (14)	4 (20)	2 (18)	2 (10)	14 (12)
Dehydration	1 (4)	4 (20)	5 (23)	0	0	3 (15)	13 (11)

^a^MedDRA System Organ Class – includes rash maculo-papular (*n* = 20, 17 %), rash macular, rash pruritic (each *n* = 15, 13 %), rash papular (*n* = 12, 10 %), rash erythematous (*n* = 11, 9 %), rash (*n* = 7, 6 %), pruritus (*n* = 5, 4 %), dermatitis acneiform, dry skin (each *n* = 2, 2 %), alopecia, circumoral edema, erythema nodosum, exfoliative rash, night sweats, petechiae, skin exfoliation, skin hyperpigmentation, swelling face, and urticaria (each *n* = 1, <1 %). Patients could have reported >1 AE

^b^High-level term, Peripheral neuropathies NEC – includes neuropathy peripheral and peripheral sensory neuropathy

*AE* adverse event, *H&N* head and neck cancer, *MTD* maximum tolerated dose, *NEC* not elsewhere classified, *NSCLC* non-small cell lung cancer, *PC* prostate cancer, *SC* subcutaneous, *STS* soft tissue sarcoma, *TPEC* tumor pharmacodynamic expansion cohort

**Table 3:** The most common (≥3 % of patients overall) drug-related grade ≥3 AEs, overall and within the dose-escalation and expansion cohorts

AE, *n* (%)	Dose-escalation cohort (*n* = 23)	MTD expansion cohorts (*n* = 93)					Total (*N* = 116)
		NSCLC (*n* = 20)	H&N (*n* = 22)	STS (*n* = 20)	PC (*n* = 11)	TPEC (*n* = 20)	
Thrombocytopenia	5 (22)	2 (10)	7 (32)	3 (15)	3 (27)	7 (35)	27 (23)
Skin and SC tissue disorders^a^	2 (9)	4 (20)	4 (18)	5 (25)	2 (18)	1 (5)	18 (16)
Fatigue	1 (4)	1 (5)	2 (9)	2 (10)	2 (18)	3 (15)	11 (9)
Dehydration	1 (4)	1 (5)	3 (14)	0	0	2 (10)	7 (6)
Lymphopenia	0	1 (5)	1 (5)	1 (5)	0	3 (15)	6 (5)
Anemia	0	2 (10)	0	0	0	1 (5)	3 (3)
Decreased platelet count	0	2 (10)	0	1 (5)	0	0	3 (3)
Dyspnea	0	1 (5)	1 (5)	0	1 (9)	0	3 (3)
Nausea	1 (4)	0	1 (5)	0	0	1 (5)	3 (3)
Peripheral neuropathies NEC^b^	1 (4)	0	0	0	1 (9)	1 (5)	3 (3)
Vomiting	1 (4)	0	0	1 (5)	0	1 (5)	3 (3)

^a^MedDRA System Organ Class – includes rash maculo-papular (*n* = 8, 7 %), rash macular (*n* = 3, 3 %), rash (*n* = 1, <1 %), rash pruritic (*n* = 4, 3 %), rash erythematous (*n* = 2, 2 %), erythema nodosum, and rash papular (each *n* = 1, <1 %). Patients could have reported >1 AE

^b^High-level term, Peripheral neuropathies NEC – includes neuropathy peripheral and peripheral sensory neuropathy

*AE* adverse event, *H&N* head and neck cancer, *MTD* maximum tolerated dose, *NEC* not elsewhere classified, *NSCLC* non-small cell lung cancer, *PC* prostate cancer, *SC* subcutaneous, *STS* soft tissue sarcoma, *TPEC* tumor pharmacodynamic expansion cohort

Treatment-related skin and subcutaneous (SC) tissue disorders (reported under the MedDRA System Organ class), which were the most common drug-related AEs overall, were observed in 61 patients (53 %). These were grade 3 in 18 patients (16 %) and included two of the DLTs. Rash had resolved by the end of the study in all but two cases. There was no grade 4 rash. Duration of rash ranged from 4 to 90 days (median 15 days). Ten patients received IV or oral corticosteroids with or without antihistamine, four patients received IV or oral antihistamines alone, and three received topical hydrocortisone. Ixazomib was discontinued permanently for one patient experiencing rash and periorbital edema; this rash resolved with concomitant medications.

Thrombocytopenia was the third most frequent drug-related AE overall (*n* = 52, 45 %) and the most common drug-related grade ≥3 AE (*n* = 27, 23 %). Median platelet count appeared to decrease during ixazomib treatment followed by recovery during the rest period of each cycle (data not shown). In total, five (4 %) patients required platelet transfusions. Drug-related PN NEC (not elsewhere classified; high-level term that included neuropathy peripheral and peripheral sensory neuropathy) was reported in 17 patients (15 %). Most cases were grade 1 (*n* = 6) or grade 2 (*n* = 8) in intensity. Grade 3 PN NEC occurred in three patients (3 %), all of whom were receiving the MTD; one had grade 1 neuropathy at screening.

Seventeen patients (15 %) discontinued ixazomib due to treatment-emergent AEs. In the dose-escalation cohort, one non-DLT-evaluable patient receiving ixazomib 1.0 mg/m^2^ discontinued due to disease progression considered unrelated to ixazomib, one patient receiving 1.76 mg/m^2^ discontinued due to the DLT of drug-related pruritic rash, and one patient receiving 2.34 mg/m^2^ discontinued due to the DLT of drug-related acute renal failure. In the NSCLC MTD expansion cohort, four patients discontinued due to: grade 3 pneumonia considered unrelated to ixazomib (*n* = 1); drug-related grade 3 pneumonitis (*n* = 1); drug-related grade 3 acute renal failure and grade 4 thrombocytopenia (*n* = 1); and drug-related grade 2 pruritic rash and grade 2 periorbital edema (*n* = 1). In the head and neck cancer cohort, five patients discontinued due to: grade 3 dyspnea (*n* = 1); grade 2 confusional state (*n* = 1); grade 5 obstructive pneumonia secondary to progressive cancer (*n* = 1); grade 5 squamous cell carcinoma (*n* = 1); and grade 2 PN (*n* = 1). Of these five AEs leading to discontinuation, only grade 3 dyspnea was considered treatment-related. In the soft tissue sarcoma cohort, two patients discontinued due to drug-related grade 3 pneumonitis (*n* = 1) and drug-related grade 3 fatigue (*n* = 1). In the prostate cancer cohort, two patients discontinued due to drug-related grade 3 ileus (*n* = 1) and drug-related grade 3 PN (*n* = 1). Lastly, one patient in the TPEC discontinued due to grade 4 brain metastases considered unrelated to ixazomib.

Drug-related serious AEs were seen in 32 patients (28 %). The most common (>2 patients overall) of these events were thrombocytopenia (*n* = 7, 6 %), nausea, vomiting and dehydration (each *n* = 4, 3 %), and fatigue (*n* = 3, 3 %). There were seven on-study deaths. Five patients died due to progressive disease, one due to obstructive pneumonia secondary to progressive cancer (as noted above), and one due to acute renal insufficiency and hypotension. None of the deaths was considered treatment-related.

### Pharmacokinetics and pharmacodynamics

In total, 32 and 36 patients were included in the pharmacokinetic and pharmacodynamic (20S blood assay) populations, respectively. The key pharmacokinetic and pharmacodynamic parameters determined for these two populations are listed in Table 4.

**Table 4:** Ixazomib geometric mean (% coefficient of variation)^a^ Plasma pharmacokinetic parameters and mean (± standard deviation)^a^ Blood pharmacodynamic (20S) parameters on day 1 and day 11

	Ixazomib dose, mg/m^2^						
	0.125	0.25	0.5	1	1.33	1.76	2.34
Pharmacokinetic parameters							
Day 1							
*n*	1	1	1	4	4	19^b^	1
C_0_, ng/mL	15.1	82.5	192.0	347 (46)	366 (38)	582 (29)	901
AUC_0–72_, h*ng/mL	NC	NC	91.6	192 (21)	391 (23)	429 (28)	620
DN C_0_, ng/mL/mg)	75.5	183	240	184 (46)	162 (29)	184 (42)	188
DN AUC_0–72_, h*ng/mL/mg	NC	NC	115	102 (19)	173 (28)	136 (29)	129
Day 11							
*n*	1	1	1	4^c^	3	15	1
C_0_, ng/mL	27.1	69	83.8	273 (23)	390 (49)	556 (58)	(869)
AUC_0–72_, h*ng/mL	NC	60.9	301	580 (40)	1160 (35)	1280 (35)	(3800)
t_½_, h	NC	NC	NC	172 (22)	145 (19)	110 (24)	90.8
DN C_0_, ng/mL	136	153	105	138 (18)	174 (39)	175 (73)	181
DN AUC_0–72_, h*ng/mL/mg	NC	135	376	295 (34)	517 (52)	402 (30)	792
Accumulation ratio	NC	NC	3.29	3.00 (49)	2.83 (25)	3.11 (23)	6.13
Pharmacodynamic parameters							
Day 1							
*n*	1	1	1	7	4^d^	19^e^	3
E_max_, % inhibition	7.7	17.9	28.2	36.9 (±19.4)	46.7 (±9.4)	61.3 (±7.9)	64.4 (±3.4)
Time to E_max_, h^e^	1.0	0.10	0.25	0.10 (0.08–0.10)	0.25 (0.1–0.25)	0.10 (0.067–0.267)	0.10 (0.083-0.183
Day 11							
*n*	1	1	1	6	3	15	3
E_max_, % inhibition	4.5	10.0	46.1	38.9 (±8.35)	54.3 (±4.1)	62.5 (±7.71)	72.5 (±4.71)
Time to E_max_, h^f^	24.0	0.083	0.1	0.1 (0.0–0.117)	0.083 (0.083–0.1)	0.10 (0.083–0.283)	0.10 (0.083-0.133)

^a^Individual values are reported in *n* < 3

^b^ *n* = 18 for AUC_0–72_ and DN AUC_0–72_

^c^ *n* = 3 for t_½_ and the accumulation ratio

^d^ *n* = 3 for AUE_0- τ_

^e^ *n* = 18 for AUE_0–72_

^f^Values shown are median (range)

*AUC*_*0–72*_ area under the plasma ixazomib concentration versus time curve from 0 to 72 h post-dose, *C*_*0*_ extrapolated immediate post-bolus concentration of ixazomib, *DN* dose-normalized, *E*_*max*_ maximum effect, *NC* not calculated, *t*_*½*_ terminal disposition phase half-life

**Fig. 1:**
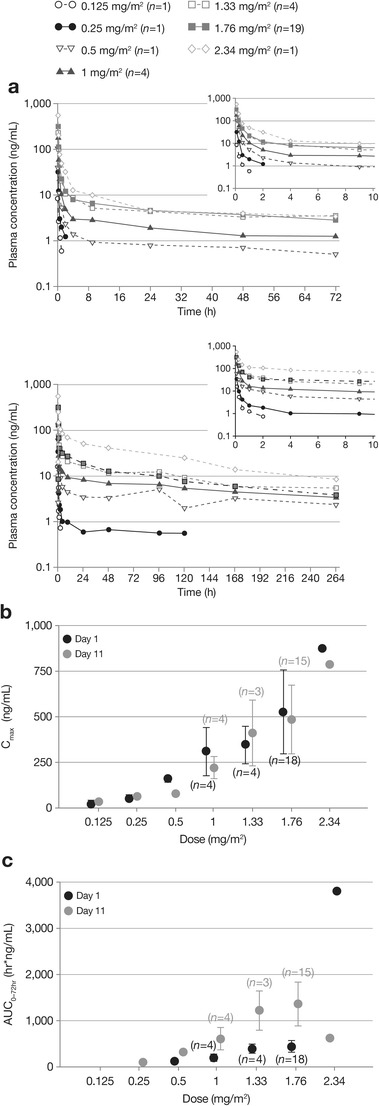
**a** Mean plasma concentration–time profiles for ixazomib on day 1 (top panel; *n* = 31) and day 11 (bottom panel; *n* = 26) of dosing, by dose level, and **b** geometric mean (% coefficient of variance) C_max_ and **c** AUC of ixazomib on day 1 and day 11 of dosing, by dose level (*n* = 23)

Mean plasma concentrations of ixazomib decreased by approximately 90 % during the initial rapid disposition phase, which lasted for approximately 8 h (Fig. 1a). The decline in plasma concentrations was then more gradual during the subsequent slow disposition phase (Fig. 1a), with the terminal half-life of ixazomib ranging from 3.8 to 7.2 days. Ixazomib plasma exposure appeared to increase proportionally with increasing ixazomib dose (from 0.5 to 2.34 mg/m^2^) (Fig. 1b and Fig. S1). At the 1.76 mg/m^2^ MTD, exposures appeared similar across the expansion cohorts. Accumulation of ixazomib was approximately 3-fold following the day 11 dose (Fig. 1c and Fig. S1).

Inhibition of 20S proteasome in whole blood was immediate (Fig. S2), and maximal inhibition correlated with maximum plasma concentration. At the MTD of 1.76 mg/m^2^, an average maximal inhibition of 20S proteasome activity of 60 % was observed. In general, activity recovered to predose levels within 24 h following single-dose administration on day 1 (Fig. S2) with the notable exception of patients treated at the dose level greater than the MTD.

**Fig. 2:**
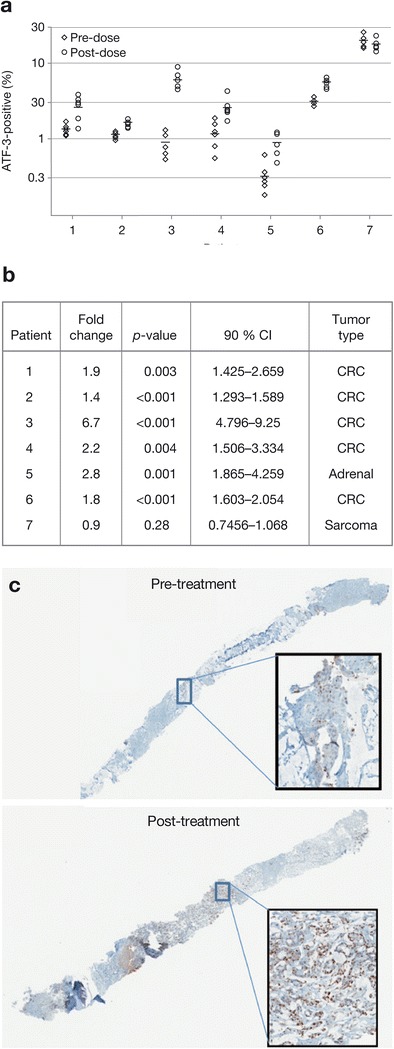
**a** Activating transcription factor-3 (ATF-3) levels in seven paired pre- and post-dose tumor biopsies from patients in the tumor pharmacodynamic expansion cohort, **b** fold-change in ATF-3 levels post- versus pre-dose and statistical significance (*t*-test) by patient, and **c** ATF-3 staining of pre- and post-dose tumor samples from one patient

For the drug distribution and ATF-3 IHC analyses, tumor biopsies were collected from all 20 patients in the TPEC. Paired pre- and post-dose biopsies of sufficient size were considered evaluable for pharmacokinetic analysis from 10 patients; ixazomib was present in all 10 post-dose biopsies analyzed. Tumor pairs from seven patients (five with colorectal cancer, one with sarcoma, and one with adrenal cancer) passed quality control by standard hematoxylin and eosin staining for tumor content, and were evaluable for ATF-3 IHC. Six of the seven paired samples showed a statistically significant increase in post-dose ATF-3 levels (*p* <0.05) (Fig. 2).

### Response to treatment

Ninety-two patients received ≥1 cycle of ixazomib treatment, had measurable disease at baseline, and had ≥1 post-baseline response assessment, and therefore were included in the response-evaluable population. Twenty-four patients were not evaluable for response due to not completing at least 1 cycle of ixazomib treatment (*n* = 12), not having measurable disease at baseline (*n* = 2), or due to an absence of post-baseline response assessment (*n* = 10). A partial response was observed in one patient with head and neck cancer. This response was achieved after 4 cycles and was maintained for 8 cycles (total duration, 6.7 months). Stable disease was recorded for 30 patients, including eight with stable disease lasting for ≥4 cycles. Among these eight patients, two were in the dose-escalation cohort, three were in the NSCLC cohort, one was in the head and neck cancer cohort and two were in the soft tissue sarcoma cohort. Measurable tumor reduction was observed in two patients with kidney malignancies. One had a 14 % reduction in tumor diameter; at the end of the study, their status was progressive disease with new lesions. The other had a 22 % reduction in tumor diameter but discontinued from the study due to grade 3 acute renal failure.

## Discussion

This first-in-human phase 1 study met its goal of defining the MTD and assessing safety of the investigational proteasome inhibitor ixazomib in patients with a spectrum of solid tumor types. The MTD for IV ixazomib on a twice-weekly schedule was established as 1.76 mg/m^2^. Treatment-related toxicities included fatigue, thrombocytopenia, gastrointestinal toxicities, and rash. The regimen was generally manageable and there was a low incidence of PN, which is consistent with observations from other studies evaluating ixazomib in patients with hematologic malignancies [26–29]. The pharmacodynamic investigations demonstrated dose-dependent 20S proteasome inhibition in blood. Dose limiting toxicity appeared to be associated with a prolonged duration of 20S proteasome inhibition in patients treated at the dose level above the MTD, although the number of patients treated is too small to make any definitive correlation. The study also provided evidence of target engagement in tumor tissue during treatment through the increase in ATF-3 expression in post-dose tumor samples. ATF-3 is a marker of unfolded protein response/endoplasmic reticulum stress, which is upregulated in response to proteasome inhibition [30–32]. The ATF-3 data therefore represent the first pharmacodynamic evidence of proteasome pathway inhibition in solid tumors following ixazomib dosing. However, despite evidence of proteasome inhibition and pathway effects, IV ixazomib demonstrated only limited antitumor activity in these study patients with advanced non-hematologic malignancies. This is consistent with reports that pharmacological response is not a guarantee of a significant clinical response [33, 34]. Measurement of drug effect at the tumor site provides direct evidence that the drug has reached its target. ATF-3 upregulation has been widely observed in preclinical experiments conducted in more than 15 xenograft tumors treated with ixazomib; however, not all these xenograft models show tumor growth inhibition in response to ixazomib, and therefore it serves as a pharmacodynamic marker and not a predictor of efficacy even in preclinical models (data not shown).

The five DLTs reported with IV twice-weekly ixazomib included two cases of grade 3 pruritic rash reported at or below the MTD, grade 3 and 4 thrombocytopenia, and grade 3 acute renal failure seen at 2.34 mg/m^2^, the dose level above the MTD. The overall toxicity profile observed in this study was similar to those previously reported in other clinical studies of oral ixazomib in MM and light-chain amyloidosis, and of IV ixazomib in lymphoma (in which rash and hematologic AEs were the most common DLTs) [26–29].

Overall, AEs were manageable; common all-grade and grade ≥3 toxicities included fatigue, thrombocytopenia, gastrointestinal toxicities, and skin toxicities, especially rash. Generally, AEs of rash were manageable with antihistamines or low-dose topical/oral corticosteroids (if they did not resolve spontaneously during the rest week of therapy), and were reversible in the majority of cases, while thrombocytopenia appeared to be cyclical and reversible, as reported in other studies of ixazomib [27, 29]. Gastrointestinal toxicities tended to resolve with appropriate supportive therapy. Comparable to other studies of single-agent ixazomib [26–29], there was a low rate of treatment-related PN, including 3 % grade ≥3 events, all at the MTD. This is in contrast to the rates seen with bortezomib, where PN is a more common toxicity [35], but it is similar to the low rates reported for carfilzomib [36, 37]. It should be noted, however, that patients received a median of 2 cycles of treatment, and only one-fifth of patients received 4 cycles or more. Therefore, this treatment exposure may not have permitted a comprehensive understanding of the safety and tolerability profile of IV ixazomib in patients with solid tumors.

This paper describes for the first time the pharmacokinetic profile of ixazomib in patients with solid tumors. The pharmacokinetic analysis showed that ixazomib plasma concentrations decreased by approximately 90 % within the first 8 h post-dose and the terminal half-life ranged from 3.8 to 7.2 days following multiple dosing, similar to results seen in patients with MM [29]. Additionally, across the full range of doses tested (0.5 to 2.34 mg/m^2^), plasma exposure appeared to increase proportionally with increasing dose, and this did not differ markedly among patients with different tumor types. Current studies are investigating the pharmacokinetic profile of ixazomib following oral dosing, which has been selected as the route of administration for further clinical development of ixazomib in patients with hematologic malignancies, where the drug shows substantial clinical activity [26–29].

The lack of clinical activity in the present study is comparable to observations from bortezomib and carfilzomib studies in advanced solid tumors [7–13, 38]. At the MTD of 1.76 mg/m^2^, only one patient with head and neck cancer achieved a partial response with ixazomib treatment. The lack of efficacy may be due to suboptimal duration of target inhibition at tolerable doses, but clinical activity can depend on many factors in addition to target inhibition, such as treatment duration, toxicity, and patient selection. A better understanding of additional response determinants including degree and duration of target inhibition is needed to improve outcomes in clinical studies investigating proteasome inhibitors. In conclusion, this study determined the safety profile, MTD, and pharmacokinetics and blood pharmacodynamics of IV ixazomib in patients with non-hematologic malignancies. Despite the demonstrated drug distribution and downstream effects of proteasome inhibition in solid tumor tissue, limited antitumor activity was observed in predefined tumor types, although it is of interest that one heavily pretreated patient with head and neck carcinoma experienced a durable response. Weekly oral ixazomib is currently being investigated in ongoing phase 3 trials in MM and primary systemic AL amyloidosis.

## Electronic supplementary material

Below is the link to the electronic supplementary material.

Supplementary Fig. 1 Dose proportionality analysis for AUC of ixazomib on day 1 (top panel; *n* = 28) and day 11 (bottom panel; *n* = 25) of dosing (TIFF 1271 kb) High Resolution (GIF 14 kb)

Supplementary Fig. 2 Mean whole blood 20S proteasome inhibition profiles following treatment with ixazomib on day 1 (top panel) and day 11 (bottom panel) of dosing, by dose level (*n* = 36) (TIFF 1740 kb) High Resolution (GIF 30 kb)

Supplementary Table 1 Overall Safety Profile of Ixazomib (DOC 568 kb)
