# Supplementary figures and images for: Phase 1 study of ixazomib, an investigational proteasome inhibitor, in advanced non-hematologic malignancies
Source: Invest New Drugs. 2015 Mar 18;33(3):652–63. doi: 10.1007/s10637-015-0230-x (PMC4435632; doi:10.1007/s10637-015-0230-x)

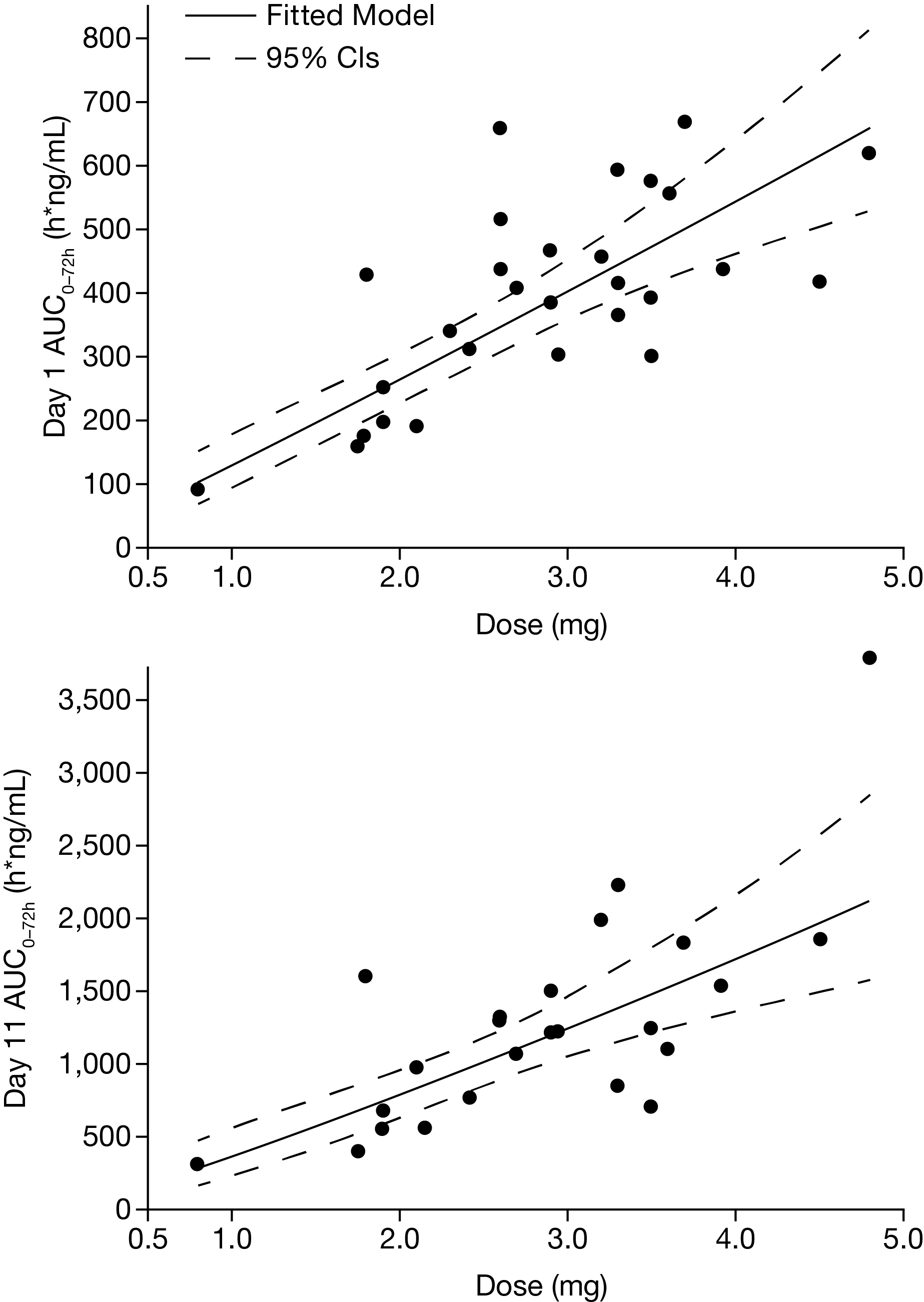

Supplement: Supplementary file 1 — Dose proportionality analysis for AUC of ixazomib on day 1 (top panel; n = 28) and day 11 (bottom panel; n = 25) of dosing (TIFF 1271 kb) [file 10637_2015_230_MOESM1_ESM.tif]

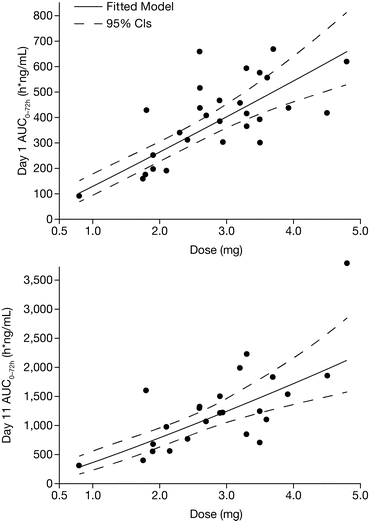

Supplement: Supplementary file 2 — High Resolution (GIF 14 kb) [file 10637_2015_230_Fig3_ESM.gif]

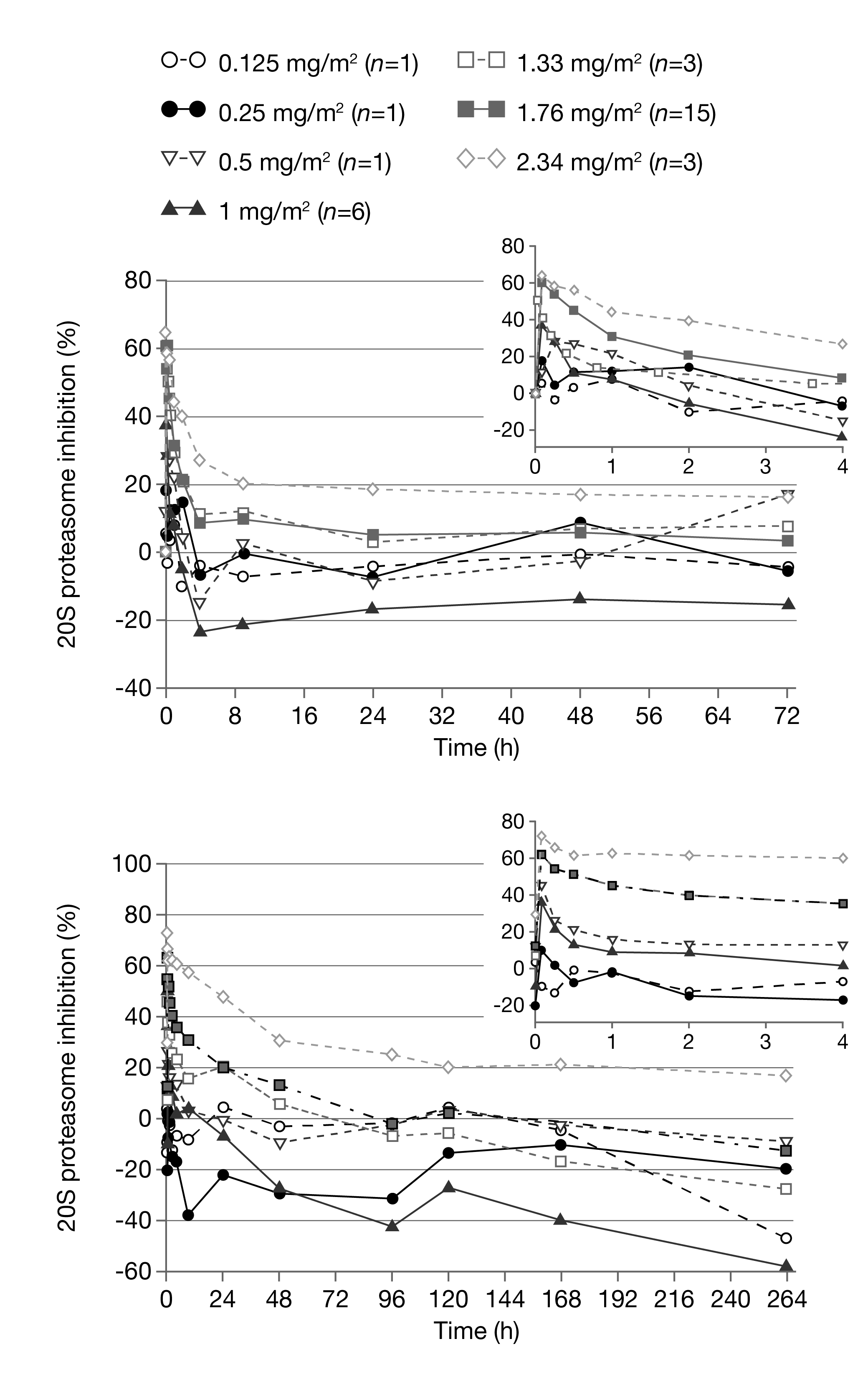

Supplement: Supplementary file 3 — Mean whole blood 20S proteasome inhibition profiles following treatment with ixazomib on day 1 (top panel) and day 11 (bottom panel) of dosing, by dose level (n = 36) (TIFF 1740 kb) [file 10637_2015_230_MOESM2_ESM.tif]

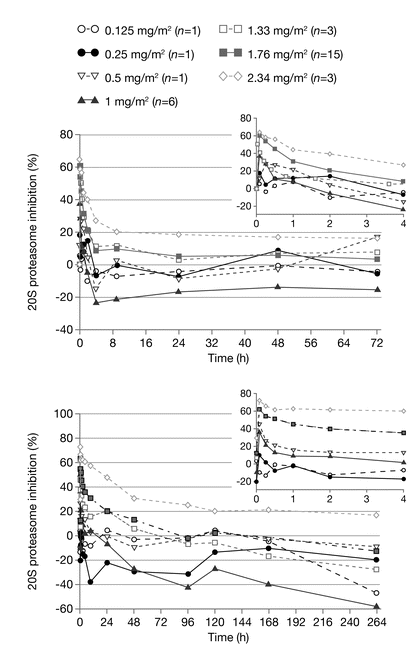

Supplement: Supplementary file 4 — High Resolution (GIF 30 kb) [file 10637_2015_230_Fig4_ESM.gif]
