# Supplementary material for: Phase 1 study of ixazomib, an investigational proteasome inhibitor, in advanced non-hematologic malignancies
Source: Invest New Drugs. 2015 Mar 18;33(3):652–63. doi: 10.1007/s10637-015-0230-x (PMC4435632; doi:10.1007/s10637-015-0230-x)
Supplement: Supplementary file 5 — Overall Safety Profile of Ixazomib (DOC 568 kb) [file 10637_2015_230_MOESM3_ESM.doc]

|  |  |  | | | | |  |
| --- | --- | --- | --- | --- | --- | --- | --- |
|  |  |  |  |  |
|  |  |  |  |  |  |  |  |
|  |  |  |  |  |  |  |  |
|  |  |  |  |  |  |  |  |
|  |  |  |  |  |  |  |  |
|  |  |  |  |  |  |  |  |
|  |  |  |  |  |  |  |  |
|  |  |  |  |  |  |  |  |
|  |  |  |  |  |  |  |  |
|  |  |  |  |  |  |  |  |
|  |  |  |  |  |  |  |  |
|  |  |  |  |  |  |  |  |
|  |  |  |  |  |  |  |  |
|  |  |  |  |  |  |  |  |
|  |  |  |  |  |  |  |  |
|  |  |  |  |  |  |  |  |
|  |  |  |  |  |  |  |  |
|  |  |  |  |  |  |  |  |
|  |  |  |  |  |  |  |  |
|  |  |  |  |  |  |  |  |

|  |  |  | | | | |  |
| --- | --- | --- | --- | --- | --- | --- | --- |
|  |  |  |  |  |
|  |  |  |  |  |  |  |  |
|  |  |  |  |  |  |  |  |
|  |  |  |  |  |  |  |  |
|  |  |  |  |  |  |  |  |
|  |  |  |  |  |  |  |  |
|  |  |  |  |  |  |  |  |
|  |  |  |  |  |  |  |  |
|  |  |  |  |  |  |  |  |
|  |  |  |  |  |  |  |  |
|  |  |  |  |  |  |  |  |
|  |  |  |  |  |  |  |  |

|  |  |  | | | | |  |
| --- | --- | --- | --- | --- | --- | --- | --- |
|  |  |  |  |  |
|  |  |  |  |  |  |  |  |
|  |  |  |  |  |  |  |  |
|  |  |  |  |  |  |  |  |
|  |  |  |  |  |  |  |  |
|  |  |  |  |  |  |  |  |
|  |  |  |  |  |  |  |  |
|  |  |  |  |  |  |  |  |
|  |  |  |  |  |  |  |  |
|  |  |  |  |  |  |  |  |
|  |  |  |  |  |  |  |  |
|  |  |  |  |  |  |  |  |

|  |  | | | | | | |
| --- | --- | --- | --- | --- | --- | --- | --- |
|  |  |  |  |  |  |  |  |
|  | | | | | | | |
|  |  |  |  |  |  |  |  |
|  |  |  |  |  |  |  |  |
|  |  |  |  |  |  |  |  |
|  |  |  |  |  |  |  |  |
|  |  |  |  |  |  |  |  |
|  |  |  |  |  |  |  |  |
|  |  |  |  |  |  |  |  |
|  |  |  |  |  |  |  |  |
|  |  |  |  |  |  |  |  |
|  |  |  |  |  |  |  |  |
|  |  |  |  |  |  |  |  |
|  |  |  |  |  |  |  |  |
|  |  |  |  |  |  |  |  |
|  |  |  |  |  |  |  |  |
|  | | | | | | | |
|  |  |  |  |  |  |  |  |
|  |  |  |  |  |  |  |  |
|  |  |  |  |  |  |  |  |
|  |  |  |  |  |  |  |  |
|  |  |  |  |  |  |  |  |
|  |  |  |  |  |  |  |  |
|  |  |  |  |  |  |  |  |
|  |  |  |  |  |  |  |  |

**Supplementary Table 1** Overall Safety Profile of Ixazomib

|  | **Dose-escalation cohort (*n*=23)** | **MTD expansion cohorts (*n*=93)** | | | | | **Total (*N*=116)** |
| --- | --- | --- | --- | --- | --- | --- | --- |
|  |  | **NSCLC (*n*=20)** | **H&N (*n*=22)** | **STS (*n*=20)** | **PC (*n*=11)** | **TPEC (*n*=20)** |  |
| Any AE, n (%) | 23 (100) | 20 (100) | 22 (100) | 19 (95) | 11 (100) | 20 (100) | 115 (99) |
| Any drug-related AE, n (%) | 18 (78) | 19 (95) | 20 (91) | 17 (85) | 11 (100) | 19 (95) | 104 (90) |
| Any grade ≥3 AE, n (%) | 15 (65) | 13 (65) | 19 (86) | 12 (60) | 11 (100) | 14 (70) | 84 (72) |
| Any drug-related grade ≥3 AE, n (%) | 8 (35) | 10 (50) | 15 (68) | 11 (55) | 10 (91) | 12 (60) | 66 (57) |
| Any serious AE, n (%) | 11 (48) | 9 (45) | 10 (45) | 7 (35) | 6 (55) | 11 (55) | 54 (47) |
| Any drug-related serious AE | 3 (13) | 6 (30) | 6 (27) | 4 (20) | 4 (36) | 9 (45) | 32 (28) |
| AE resulting in discontinuation, n (%) | 3 (13) | 4 (20) | 5 (23) | 2 (10) | 2 (18) | 1 (5) | 17 (15) |
| Any on-study deaths, n (%) | 3 (13) | 0 | 2 (9) | 1 (5) | 0 | 1 (5) | 7 (6) |

AE adverse event, H&N head and neck cancer, MTD maximum tolerated dose, NSCLC non-small cell lung cancer; PC prostate cancer, STS soft tissue sarcoma, TPEC tumor pharmacodynamic expansion cohort
